# A randomized comparison between three types of irrigating fluids during transurethral resection in benign prostatic hyperplasia

**DOI:** 10.1186/1471-2253-10-7

**Published:** 2010-05-28

**Authors:** Ayman A Yousef, Ghada A Suliman, Osama M Elashry, Mahmoud D Elsharaby, Abd El-naser K Elgamasy

**Affiliations:** 1 17 El-emam Moslem street, Tanta, Egypt

## Abstract

**Background:**

Central nervous system changes, circulatory and electrolyte imbalances are the main complications of endoscopic transurethral resection of the prostate (TURP) which is known as transurethral resection (TUR) syndrome, which occurs as result of excessive absorption of irrigating fluid. We compare glycine 1.5% versus glucose 5% and normal saline 0.9% as irrigating solutions during TURP in patients with moderate to severe bladder outlet obstruction due to benign prostatic hyperplasia (BPH).

**Methods:**

Three hundred sixty patients with symptomatic BPH were randomized into a prospective, controlled trial comparing the three irrigation modalities. One-hundred twenty patients used glycine 1.5% solution as irrigating fluid (glycine group), 120 patients used glucose 5% solution (glucose group) and 120 patients used normal saline 0.9% solution (saline group). Patient's demographics, operation time, hospital stay, postoperative amino acid glycine assay, postoperative serum cardiac troponin I and perioperative complications were noted.

**Results:**

No difference was found between the groups in the immediate postoperative levels of hemoglobin and hematocrite. A high glycine level was associated with the TUR syndrome. Seventeen patients had TUR syndrome; all were in glycine group and they had the highest postoperative amino acid glycine levels. Slight increase in serum sodium (142.6 ± 12.6 mmol/l) was detected in saline group. Transient Hyperglycemia (170 ± 35.9 mg/dl) and hypokalemia (3.67 ± 0.92 mmol/l) occurred in the immediate postoperative period in the glucose group.

**Conclusion:**

Endoscopic TURP performed using either glucose 5% or saline 0.9% irrigating solution during and after surgery is associated with lower incidence of TUR syndrome, lower catheterization period, shorter hospital stay and no cardiac toxicity in comparison with glycine 1.5% solution.

**Trial Registration:**

This clinical trail had been approved and registered in PACT Registry; with identification number for the registry is ATMR2010010001793131.

## Background

Many endoscopic surgical procedures require the use of an irrigating fluid to dilate the operating field and to wash away debris and blood. A potential complication of such irrigation is a systemic absorption of the fluid to the extent that overt symptoms are produced [1]. The ideal irrigant for endoscopic resection would be a user-friendly, non-conductor medium that does not interfere with diathermia, has a high degree of translucency, has similar osmolarity to the serum and causes only minimal side effects when absorbed [2]. There are several different irrigating fluids available commercially and it may be difficult to know which one to use. The choice tends to be governed largely by tradition, although the price and properties of the fluid (e.g. stickiness and transparency) also play a role. The pharmacological effects of the fluid become important whenever it is absorbed by the patient. Glycine is an endogenous amino acid without an allergic reaction potential. It is transparent and reasonably inexpensive. However, the solution is unphysiological because it lacks electrolyte and excessive absorption is a recognized complication [3]. Nausea, vomiting, confusion and arterial hypotension occur significantly more often when between 1.000 and 2.000 ml of glycine solution are absorbed. Severe forms of TUR syndrome are more rare but they require treatment in the intensive care unite (ICU) at least over night. The incidence and severity of symptoms of TUR syndrome increase progressively as more glycine solution is absorbed during TURP and the severity of symptoms is markedly aggravated when more than 3,000 ml are absorbed [4]. Deaths have been reported in patients undergoing TURP. Laboratory studies in animals showed that glycine has direct and indirect cardiotoxic effects [5].

Unlike glycine, glucose is a physiological solution that is readily metabolized when absorbed in most patients [6]. The aim of this study is to compare perioperative morbidity, operation time, and length of hospital stay for glycine 1.5% versus glucose 5% and saline 0.9% as irrigating solutions during TURP in patients with moderate to severe bladder outlet obstruction due to benign prostatic hyperplasia (BPH).

## Methods

**Figure 1:**
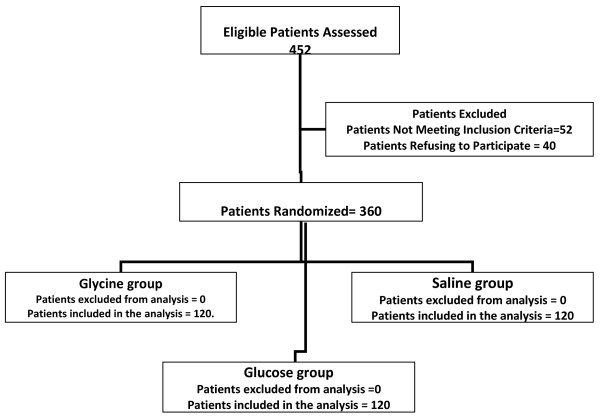
**Schematic presentation of patient flow through out trail period**.

After the study was approved by an Investigational Review Board of Faculty of Medicine, Tanta University, an informed consent was obtained from patients participating in the study. Randomization was performed by computer-generated random allocations sequence by simple randomization. A total of 360 patients undergoing TURP for BPH at Urology Department, Tanta University Hospitals were included in the study. Patients were divided into three groups according to the irrigating fluid used and randomly allocated to use either glycine 1.5% solution (glycine group, n = 120), glucose 5% (glucose group, n = 120) or normal saline 0.9% solution (saline group, n = 120) as irrigating fluid during and immediately after TURP (Figure 1). TURP was performed using 24 Ch continuous irrigating resectoscope (Storez, Tottling, Germany). Patients in saline group used bipolar loop (Storez, Tottling, Germany) as a working element for bipolar current, while the other two groups used 24Ch cutting loop and 24Ch roller loop as a working element for resectoscope for monopolar current (Storez, Tottling, Germany).

The operating room nurse assisting in the procedure randomized the patients and prepared the irrigating fluids appropriately, also enrolled the participants and assigned participants to their respective groups. All patients had been designed to receive spinal anesthesia in the form of 2.5 ml 0.5% hyperbaric bupivacaine mixed with fentanyl 20 μg intrathecally, commencement of surgery is allowed when adequate sensory block to T 10 at the umbilical level was achieved. Surgical intervention was performed by surgeons of the same qualification and clinical experience.

Evaluation of the patients included complete medical history, ultrasound for abdomen and pelvis, routine laboratory investigations (complete blood count, blood urea nitrogen, blood sugar, serum sodium, potassium, prothrombin time, albumin) and prostatic specific antigen (PSA). Immediate preoperative as well as postoperative hemoglobin, hematocrite, serum sodium and potassium, blood urea, serum creatinine, random blood glucose, serum osmolarity, arterial blood gas, as well as serum troponin-I as a cardiac cell injury marker were measured. No patients had received colloid, plasma products, hypertonic saline, diuretic therapy or blood transfusion approximately 10 hours before surgery.

Exclusions criteria included patients with bleeding disorders or existing coagulopathy, diabetes mellitus or other metabolic acidosis and apparent cardiac disease with ECG evidence of ischemia, history of myocardial infarctions and congestive cardiac failure, renal insufficiency as well as any contraindication to spinal anesthesia. All patients were pre-loaded with 500 ml ringer solution one hour before induction of spinal anesthesia. No patients received intravenous glucose or glucose saline before, during or immediately after surgical procedure. Central venous pressure catheter was inserted just before surgery to judge the status of the intravascular volume and trans-compartmental fluid shift.

Hemodynamic monitoring including: heart rate (HR), electrocardiogram (ECG), mean arterial blood pressure (MABP) and central venous pressure (CVP) were recorded. Hypotension, defined as 20% fall in blood pressure from pre-induction levels or a systolic blood pressure lower than 100 mmHg, was treated immediately by intravenous injecting of 5-10 mg ephedrine. The amount of irrigation fluids used in each patient is calculated depending upon gravimetric methods and the height of the irrigating fluid reservoir is fixed at 60 cm height from patients' bed.

TUR syndrome was defined as sodium of 125 mmol/l or less after TURP with 2 or more symptoms or signs of TUR syndrome such as nausea, vomiting, bradycardia, hypotension, chest pain, mental confusion, anxiety, parasthesia and visual disturbance [7]. Operative details including operation time, resected tissue weight, irrigating volume used, evidence of prostatic capsule perforation, catheterization time, duration of hospital stay as well as any perioperative complication were recorded. The medical and nursing stuff involved in patients care, monitoring in the post-operative period and assessment of the complications and the incidence and the severity of TUR syndrome were completely blinded to the patient's group assignment and the type irrigating fluid used.

### Glycine assay using thin layer chromatography

Thin layer chromatography is semi-quantitative method for amino acid glycine separation and assay. The principle of separation depends on differences in both the degree of adsorption by the adsorbent and solubility in the solvent used for separation, using a uniform thin layer of adsorbent on a supporting glass plate then plates are dried in an oven at 100-120°C [8].

### Troponin I assay

It is a qualitative membrane fixed immunoassay for the detection of calcium troponin I (cTnI) in whole blood, serum or plasma. The membrane is pre-coated with capture reagent in the test line regions. During the test, the serum or plasma sample react with the particle coated anti-c TnI antibodies. The mixture migrates upward on the membrane chromatographically by capillary action to react with capture reagent on the membrane and generate a colored line according to the manufacturer guide (ACON Laboratories, Inc. San Diego, USA). The presence of this colored line in the test line region indicates a positive result; while its absence indicates negative results [9].

### Statistics

Continuous parametric data variables are reported as mean ± SD and were analyzed with analysis of variance, while categorical and non-parametric variables were analyzed using x_2 _tests. A p value < 0.05 was considered significant. Based on a previous study of fluid irrigation of TUR syndrome, considering a 0.05 2-sided significance level, a power of 80%, and allocation ratio of 1:1, and allowing for 10% attrition/non-compliance rate, a group size contains 100-120 patients in each group were estimated to be sufficient [2,6].

**This clinical trail had been approved and registered in **PACT Registry; with identification number for the registry is ATMR2010010001793131.

## Results

The age of the patients ranged from 53 to 70 years old (60.7 ± 5.1) in the glycine group and 55-71 years old (60.9 ± 4.9) in the glucose group while it was 50-67 years old (62 ± 6.5) in the saline group. The operation time range was 45-70 min in glycine group (57.1 ± 8.2) and it was 40-75 min (58.3 ± 10.8) minutes in glucose group while it was 55-80 min (62.5 ± 11.2) in saline group. The mean amount of prostatic tissue resected was 89.16 gm ± 18.3(range70 to 125) gm in the glycine group and 91.9 gm ± 16 (range 75 to 120) gm in the glucose group while it was 82.5 gm ± 15.5 in saline group (range70-110). Only12 resections in glycine group and 8 in glucose group the amount of tissue resected exceed or equal to 120 gm. The indwelling catheter was removed after 2.4 ± 0.71 days (range 2 to 4) in the glycine group and after1.67 ± 0.45 days (range1 to 2) in the glucose group while after 1.54 ± 0.34 (range1 to2) in saline group. Hospital stay was 3.31 ± 0.63 days (range3 to 5) in glycine group and 2.29 ± 0.46 days (range 2 to 3) in glucose group, while it was 2.19 ± 0.38 days in saline group (range 2 to 3). (Table 1)

**Table 1:** Patients characteristic of studied groups. (Means ± SD)

	Glycine group (n = 120)	Glucose group (n = 120)	Saline group (n = 120)
**Age (years)**	60.7 ± 5.1	60.9 ± 4.9	62 ± 6.5

**Operative time (min)**	57.1 ± 8.2	58.3 ± 10.8	62.5 ± 11.2

**Resection wt (gm)**	89.16 ± 18.3	91.94 ± 16	82.5 ± 15.5

**Catheterization time (days)**	2.4 ± 0.71	1.67 ± 0.45	1.54 ± 0.34

**Hospital stay (days)**	3.31 ± 0.63	2.29 ± 0.46	2.19 ± 0.38

Analysis of variance tests were used.

The hemodynamic changes regarding heart rate (HR), mean arterial blood pressure (MABP) and central venous pressure (CVP) were compared between groups. There was no significant difference in the preoperative mean value of HR (beat/min) between the studied groups. Ten minutes after induction of anesthesia, there was a significant decrease in the mean value of HR (57.5 ± 12.6 beat/min & 56.4 ± 13.5 beat/min & 54.6 ± 11.9 beat/min) in the glycine, glucose and saline groups respectively. Then, no significant change was found through out the intra-operative and immediate postoperative period in the studied groups.

There was no significant difference in the preoperative average MABP (mmHg) between the studied groups. Ten minutes after induction of anesthesia, there was a significant decrease in the average MABP (71.6 ± 19.6 mmHg & 73.4 ± 18.5 mmHg & 72.5 ± 18.8 mmHg) in the glycine, glucose and saline groups respectively. Then, No significant change in the average MABP was found in the studied groups through out the study period.

The mean value of CVP in the studied groups was similar in the preoperative period in the studied groups. Then, significant decrease in the mean value (3.26 ± 0.95 cm/H_2_O & 3.1 ± 0.85 cm/H_2_O & 3.3 ± 0.7 cm/H_2_O) occurred 10 min after induction of anesthesia in the glycine, glucose and saline groups respectively. After 20 and 30 min, no significant change was found in the studied groups, however, significant increase in the mean value of CVP was measured at 60 min to mean value of (8.5 ± 2.4 cm/H_2_O & 8.4 ± 2.12 cm/H_2_O & 9.2 ± 2.6 cm/H_2_O) and (9.5 ± 2.54 & 9.4 ± 2.15 cm/H_2_O & 10.2 ± 2.95 cm/H_2_O) in the postoperative period in the studied groups respectively. (Table 2)

**Table 2:** Homodynamic changes in the studied groups. (Means)

	**Pre-op Gly/Glu/Sal**	**10 min Gly/Glu/Sal**	**20 min Gly/Glu/Sal**	**30 min Gly/Glu/Sal**	**60 min Gly/Glu/Sal**	**post-op Gly/Glu/Sal**
	
**HR**	62.4/64.2/63.6	57.5*/56.4*/54.6*	63.2/63/62.6	64.1/64/63.6	60.26/61.5/64.5	60.9/61.8/65.2
	
**MABP**	90.3/91.5/92.6	71.6*/73.4*/72.5*	87.9/88.8/94	89.2/91.2/96.4	91.6/95.8/98.2	93.2/96.1/99.4
	
**CVP**	5.15/5.3/5.9	3.26*/3.1*/3.3*	5.6/5.09/5.2	6.66/6.4/5.9	8.5*/8.4*/9.2*	9.5*/9.4*/10.2*

**Gly/Glu/Sal**: Glycine/Glucose/Saline groups, **MABP**: mean arterial blood pressure (mmHg), **CVP**: central venous pressure (cm/H_2_o), **HR**: heart rate (beat/m).

*Statistically significance (p < 0.05%).

Analysis of variance tests were used.

There was no significant difference in the mean value between the studied groups regarding the preoperative hemoglobin, serum sodium, serum potassium and random blood sugar. Insignificant decrease in the postoperative serum sodium was observed in glycine and glucose groups, while insignificant increase was observed in saline group (142.6 ± 12.6 mmol/l). Insignificant reduction in serum potassium in glycine and saline group was observed, but more pronounced decrease in glucose group (3.67 ± 0.92 mmol/l) was measured postoperatively. There was a significant elevation in the postoperative mean value of blood sugar level in the glucose group (170.2 ± 35.9 mg/dl) which returned back to normal level 6 hours postoperatively. (Table 3)

**Table 3:** Chemical and hematological values of studied groups in the immediate postoperative period. (Means ± SD)

	Glycine group (n = 120)	Glucose group (n = 120)	Saline group (n = 120)
Hemoglobin (gm/dl)	11.1 ± 1	10.9 ± 9	11.4 ± 1.2

Sodium (mmol/l)	134.7 ± 13.4	135.5 ± 12.9	142.6 ± 12.6

Potassium (mmol/**l)**	3.87 ± 1.17	3.67 ± 0.92	4.16 ± 1.32

Random blood sugar (mg/dl)	113.5 ± 25.5	170.2* ± 35.9	116.8 ± 28.4

*Statistically significance (p < 0.05%).

Analysis of variance tests were used.

Two Patients in glycine and another 2 patients in glucose group needed blood transfusion, who experienced a decrease in hemoglobin concentration to less than 9 g/dl. TUR syndrome developed in 17 patients in the glycine group but non in neither glucose nor saline groups. Elevated glycine levels was observed in 36 patients in glycine group of whom the highest 17 values suffered TUR syndrome. Six patients in the glycine group developed ischemic ECG changes. Three patients in glycine group developed elevated troponin I. These patients admitted to post anesthesia care unite for proper treatment. (Table 4)

**Table 4:** Peri-operative complications in the studied groups.

	Glycine group (n = 120)	Glucose group (n = 120)	Saline group (n = 120)
TUR syndrome	17*	0	0

ECG changes	6	0	0

Elevated glycine	36*	0	0

Elevated tropnin-I	3	0	0

Clot retention	0	0	1

Blood transfusion	2	2	0

Urinary retention	0	0	0

Statistically significance (p < 0.05%).

X_2 _test was used.

TUR syndrome: trans-urethral resection syndrome.

## Discussion

This randomized single blinded trail was performed in patients with prostatic hyperplasia admitted for endoscopic resection of the prostate using three different types of irrigating fluids during resection, demonstrated high incidence of TUR syndrome in patients used glycine 1.5% solution, while non in neither glucose nor saline groups developed TUR syndrome. Elevated glycine levels was observed in patients in glycine group of whom the highest values suffered TUR syndrome and was associated with ischemic ECG changes and elevated troponin I in these patients.

The use of an irrigating fluid during many endoscopic surgical procedures is mandatory to dilate the operating field and to wash away debris and blood. The systemic absorption of such an irrigating fluid may be associated with serious complications. Large-scale fluid absorption is rare but leads to symptoms severe enough to require intensive care. Patho-physiological mechanisms consist of pharmacological effects of irrigant solutes, the volume effect of irrigant water, dilutional hyponatraemia and brain edema [1].

Glycine solution is the most commonly used irrigant in TURP. Many studies performed on human denoting that glycine absorption causes echocardiogram changes and it is associated with increased troponin I [6]. Another experimental studies showed that glycine has a cardio-toxic properties and fluid absorption during TURP has devitalizing effect on the heart [10]. High glycine levels are suspected of causing cerebral edema [11], visual disturbances and even transient blindness [12,13].

Hyper-ammonaemic encephalopathy may develop as ammonia is an intermediate product in glycine metabolism [14]. Another disorder of glycine metabolism characterized by episodes of ketosis and metabolic acidosis that may proceed to coma had been reported [15]. Potentially safer alternatives to glycine irrigation are normal saline 0.9% and glucose 5% to be used as irrigating fluid during TURP. Normal saline is the ideal irrigation fluid for TURP; however its electrical conducting properties prohibit its use with conventional monpolar TURP system in the past. The advance of using bipolar resectoscope that allows resection using normal saline allows us to use it safely with no risks of precipitating hyponatreamia which is the main pathology in TUR syndrome. However, it is rapid infusion of normal saline 0.09% that can cause hypercholeramic metabolic acidosis [7,11].

Glucose 5% is relatively more physiological than glycine because it can be given intravenously and with lower incidence of complication. A solution of glucose 5% is metabolized throughout the body, it requires 13 L to be given/absorbed intravenously to expand the intravascular compartment by 1 L [2].

Normal serum osmolality is ≈ 290 mOsm/L. The osmolality of normal saline 0.9% is about 300 mOsm/L, and that of glucose 5% is 285 mOsm/L, as opposed to the osmolality of glycine 1.5%, which is 190 mOsm/L. This higher osmolality provided by both normal saline 0.9% and glucose 5% solution may be beneficial in reducing the possible side effects of cerebral edema.

Issa et al., [11] in a case study concluded that bipolar saline is a safe and eliminates the risk of TUR syndrome in high-risk patients with large prostates. Michielsen et al., [7] concluded in his study that a bipolar transurethral resection in saline system is as efficacious as monopolar transurethral prostate resection but it is safer than the latter because of the lesser changes in post-operative sodium, and the smaller risk of transurethral resection syndrome.

Two studies done by Collins et al, [2,6] the first study concluded that an increase in serum glycine was associated with TUR syndrome; there were large variations in the amounts of glycine absorbed, reaching levels many times the upper limit of normal, and although there was immediate postoperative hyperglycemia in patients used glucose 5% as irrigation fluid during TURP, it was not associated with either ECG changes nor elevated serum troponin I. In the second study, glycine was reportedly toxic, producing ECG changes and increase in the serum troponin I. Unrecognized blood loss or glycine absorption may explain the increase in morbidity and mortality reported in patients who undergo TURP.

## Conclusion

Endoscopic transurethral of the prostate performed using either bipolar normal saline 0.9% resection, or monoplar glucose 5% resection as irrigating solution during and after surgery, when compared with monoplar glycine 1.5% resection, are associated with lower perioperative morbidity including TUR syndrome, lower catheterization period and shorter hospital stay. Except for the transient postoperative hyperglycemia, in glucose group, both systems are nearly equivalent.

## Abbreviations

TURP: Transurethral resection of the prostate; CVP: Central venous pressure; TUR Syndrome: Transurethral resection syndrome; HR: Heart rate; BPH: Benign prostatic hyperplasia; PSA: Prostatic specific antigen; C Tn I: Calcium troponin I; ECG: Electrocardiogram; MABP: Mean arterial blood pressure.

## Competing interests

The authors declare that they have no competing interests.

## Authors' contributions

AAY performed the anesthetic management, prepared the manuscript, and patients follow up. O M Elashry, M D Elsharaby   and A K Elgamasy   performed the surgical intervention and   patients follow up. GAS prepared the lab results and assessed in manuscript preparation. All authors read and approved the final manuscript.

## Authors' Details

Ayman A Yousef.

Lecturer of Anesthesiology, Department of Anesthesia, Tanta University Hospitals, El-Geish street, Tanta, 31527, Egypt.

Ghada A Suliman.

Lecturer of Clinical Pathology, Department of Clinical Pathology, Tanta University Hospitals, El-Geish street, Tanta, 31527, Egypt.

Osama M Elashry.

Assistant professor of Urology, Department of Urology, Tanta University Hospitals, El-Geish street, Tanta, 31527, Egypt.

Mahmoud D Elsharaby.

Professor of Urology, Department of Urology, Tanta University Hospitals, El-Geish street, Tanta, 31527, Egypt.

Abd El-naser K Elgamasy.

Professor of Urology, Department of Urology, Tanta University Hospitals, El-Geish street, Tanta, 31527, Egypt.

## Pre-publication history

The pre-publication history for this paper can be accessed here:

http://www.biomedcentral.com/1471-2253/10/7/prepub
